# Supplementary material for: Self-cleaving peptides for expression of multiple genes in Dictyostelium discoideum
Source: PLoS One. 2023 Mar 2;18(3):e0281211. doi: 10.1371/journal.pone.0281211 (PMC9980757; doi:10.1371/journal.pone.0281211)
Supplement: S1 Raw images — (PDF) [file pone.0281211.s001.pdf]

Western Blot supporting Fig 1B,C
Chemiluminescence overlaid on membrane
(raw files with chemiluminescence channel separate from membrane available on Dryad repository)

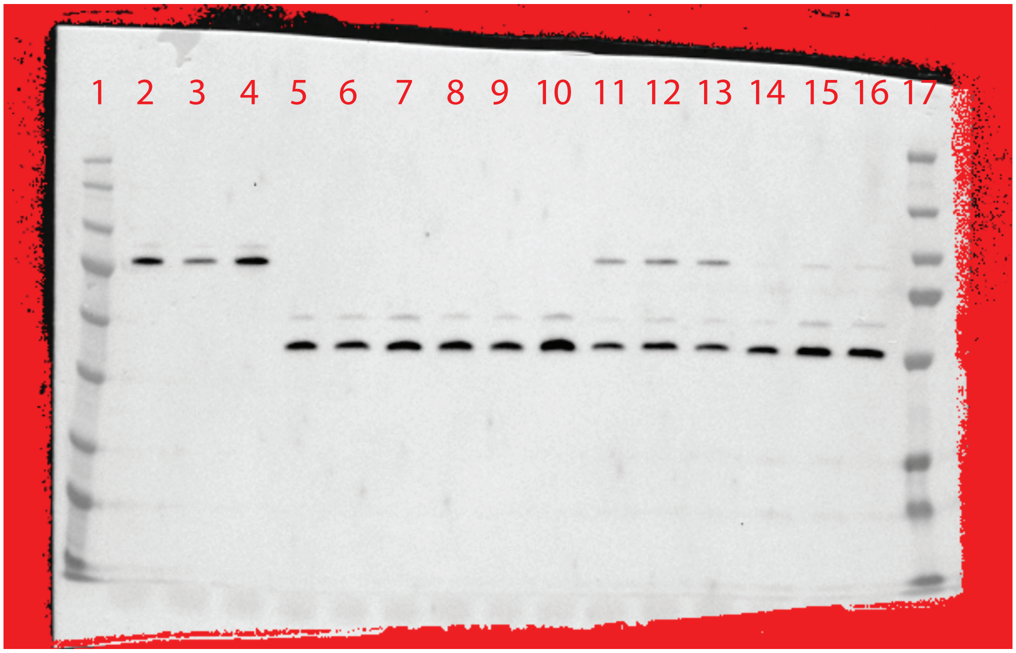

No-Stain Protein Labeling Reagent overlaid on membrane
(raw files with fluorescence channel separate from membrane available on Dryad repository)

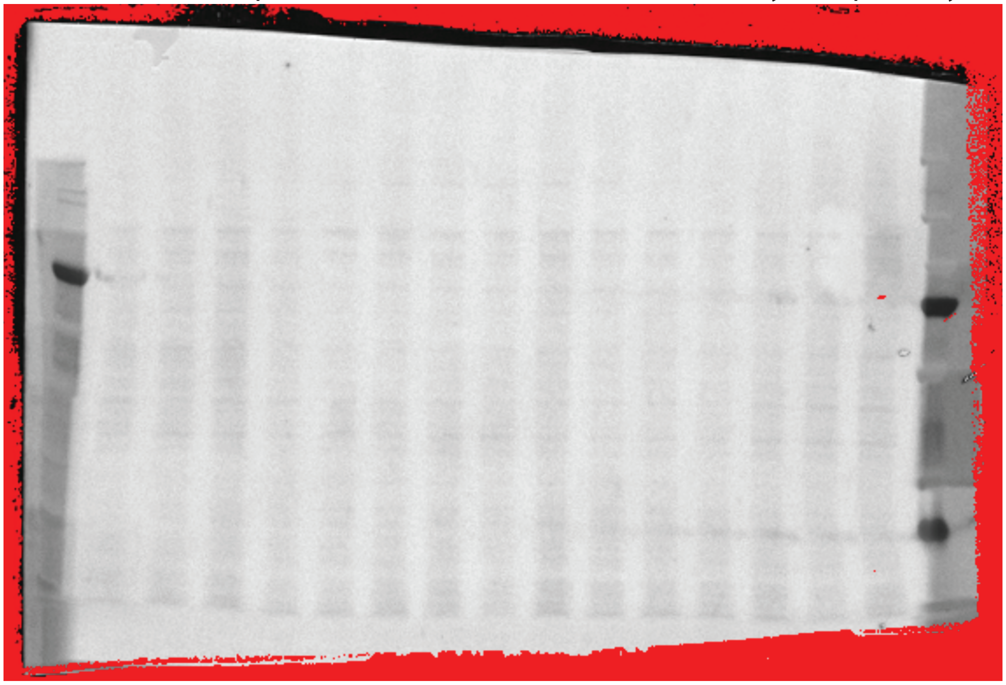

Blot loaded as follows:
1) Flash Protein Ladder, 10-180 kDa FPL-006 (Gel Company)
2-4) mCherry-H2B-linker-mNeonGreen
5-7) mCherry-H2B-P2A-mNeonGreen
8-10) mCherry-H2B-T2A-mNeonGreen
11-13) mCherry-H2B-F2A-mNeonGreen
14-16) mCherry-H2B-E2A-mNeonGreen
17) PageRuler Plus Prestained Protein Ladder (ThermoFisher Scientific)
