# Supplementary figures and images for: Self-cleaving peptides for expression of multiple genes in Dictyostelium discoideum
Source: PLoS One. 2023 Mar 2;18(3):e0281211. doi: 10.1371/journal.pone.0281211 (PMC9980757; doi:10.1371/journal.pone.0281211)

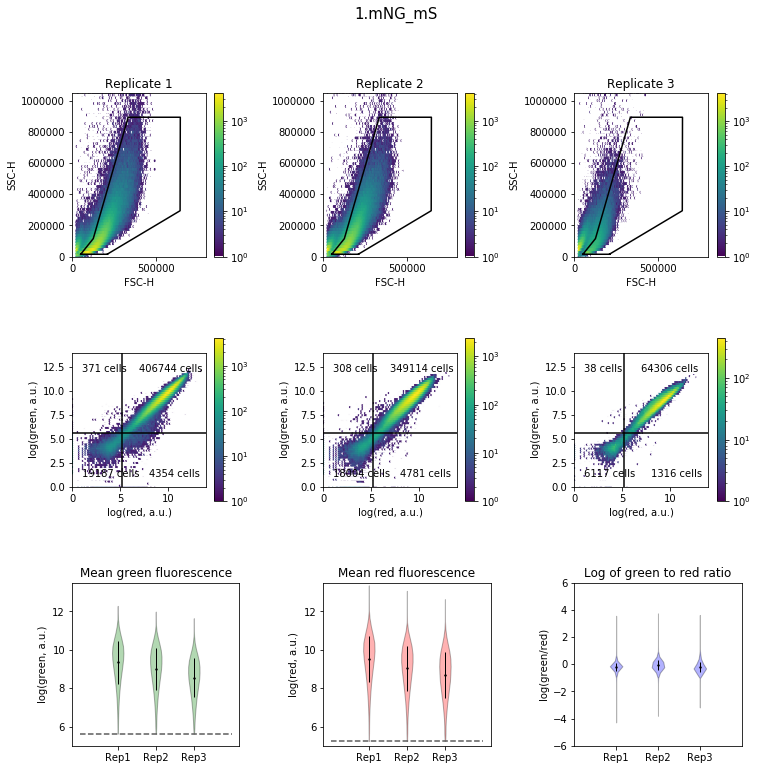

Supplement: S3 Fig — Top: Forward scatter and side scatter profiles of all three replicates. Density map color indicates event count. Events inside the polygon, defined as shown in S2 Fig, were considered live cells. Middle: Green and red fluorescence values for live cells for each replicate. Displayed numbers indicate total event count in a given quadrant. Cells in the upper right quadrant were retained for further analysis. Bottom: Mean fluorescence values and ratio of green to red fluorescence for each replicate. For each violin plot of single-cell values, the overlaid dot represents mean of the distribution and the error bars represent the standard deviation. Dashed lines represent fluorescence thresholds. (TIFF) [file pone.0281211.s006.tiff]

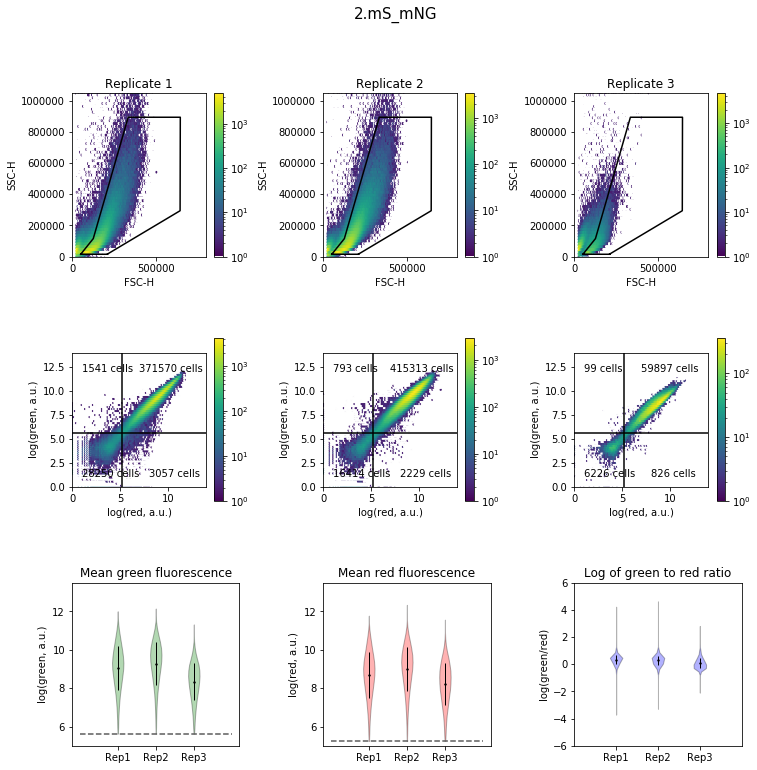

Supplement: S4 Fig — Top: Forward scatter and side scatter profiles of all three replicates. Density map color indicates event count. Events inside the polygon, defined as shown in S2 Fig, were considered live cells. Middle: Green and red fluorescence values for live cells for each replicate. Displayed numbers indicate total event count in a given quadrant. Cells in the upper right quadrant were retained for further analysis. Bottom: Mean fluorescence values and ratio of green to red fluorescence for each replicate. For each violin plot of single-cell values, the overlaid dot represents mean of the distribution and the error bars represent the standard deviation. Dashed lines represent fluorescence thresholds. (TIFF) [file pone.0281211.s007.tiff]

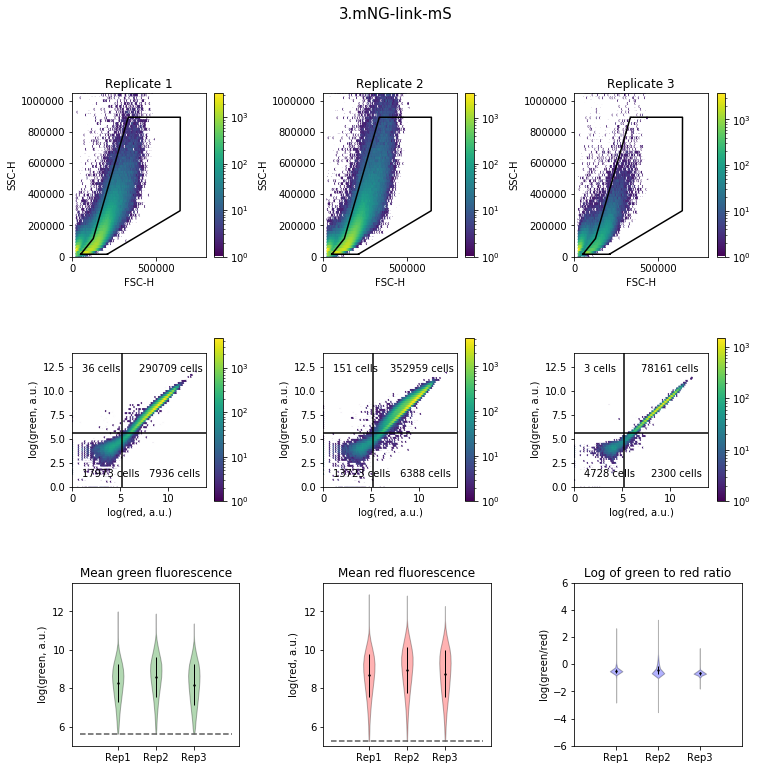

Supplement: S5 Fig — Top: Forward scatter and side scatter profiles of all three replicates. Density map color indicates event count. Events inside the polygon, defined as shown in S2 Fig, were considered live cells. Middle: Green and red fluorescence values for live cells for each replicate. Displayed numbers indicate total event count in a given quadrant. Cells in the upper right quadrant were retained for further analysis. Bottom: Mean fluorescence values and ratio of green to red fluorescence for each replicate. For each violin plot of single-cell values, the overlaid dot represents mean of the distribution and the error bars represent the standard deviation. Dashed lines represent fluorescence thresholds. (TIFF) [file pone.0281211.s008.tiff]

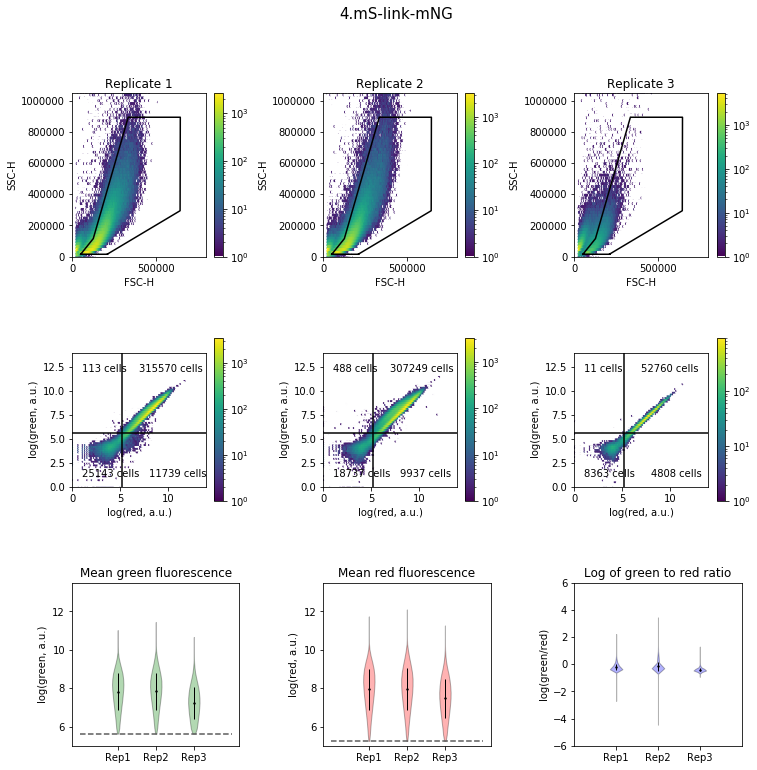

Supplement: S6 Fig — Top: Forward scatter and side scatter profiles of all three replicates. Density map color indicates event count. Events inside the polygon, defined as shown in S2 Fig, were considered live cells. Middle: Green and red fluorescence values for live cells for each replicate. Displayed numbers indicate total event count in a given quadrant. Cells in the upper right quadrant were retained for further analysis. Bottom: Mean fluorescence values and ratio of green to red fluorescence for each replicate. For each violin plot of single-cell values, the overlaid dot represents mean of the distribution and the error bars represent the standard deviation. Dashed lines represent fluorescence thresholds. (TIFF) [file pone.0281211.s009.tiff]

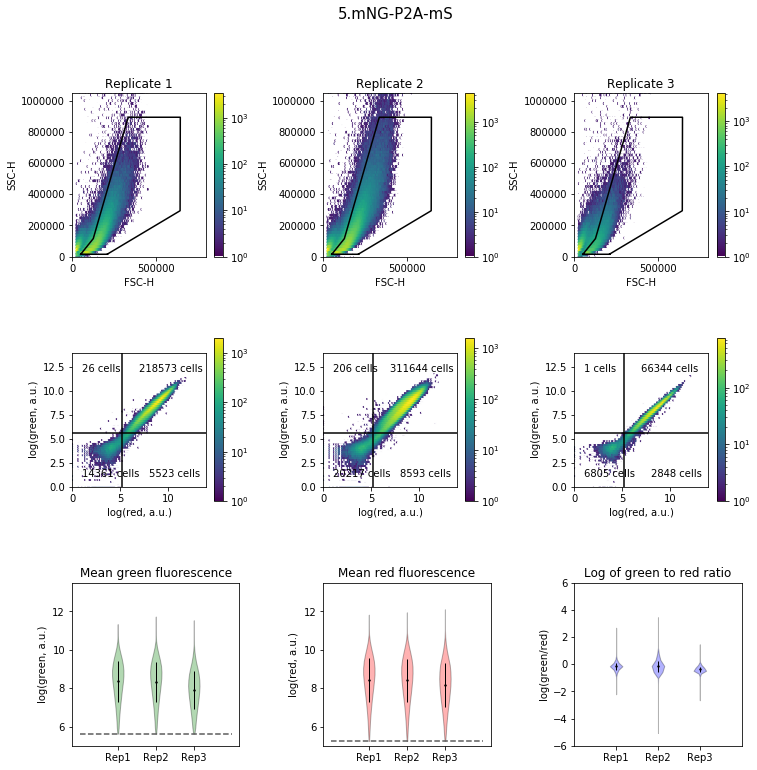

Supplement: S7 Fig — Top: Forward scatter and side scatter profiles of all three replicates. Density map color indicates event count. Events inside the polygon, defined as shown in S2 Fig, were considered live cells. Middle: Green and red fluorescence values for live cells for each replicate. Displayed numbers indicate total event count in a given quadrant. Cells in the upper right quadrant were retained for further analysis. Bottom: Mean fluorescence values and ratio of green to red fluorescence for each replicate. For each violin plot of single-cell values, the overlaid dot represents mean of the distribution and the error bars represent the standard deviation. Dashed lines represent fluorescence thresholds. (TIFF) [file pone.0281211.s010.tiff]

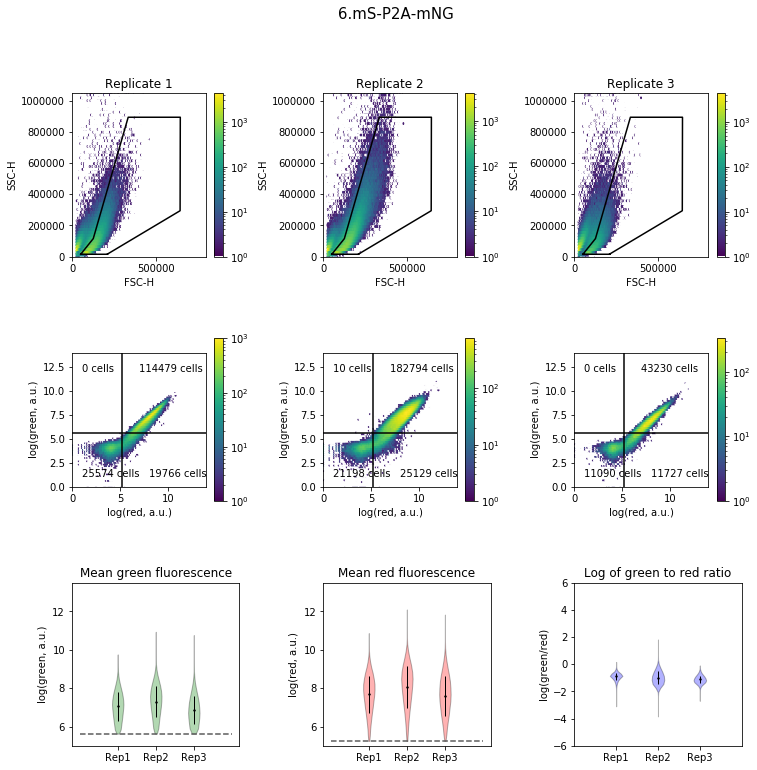

Supplement: S8 Fig — Top: Forward scatter and side scatter profiles of all three replicates. Density map color indicates event count. Events inside the polygon, defined as shown in S2 Fig, were considered live cells. Middle: Green and red fluorescence values for live cells for each replicate. Displayed numbers indicate total event count in a given quadrant. Cells in the upper right quadrant were retained for further analysis. Bottom: Mean fluorescence values and ratio of green to red fluorescence for each replicate. For each violin plot of single-cell values, the overlaid dot represents mean of the distribution and the error bars represent the standard deviation. Dashed lines represent fluorescence thresholds. (TIFF) [file pone.0281211.s011.tiff]

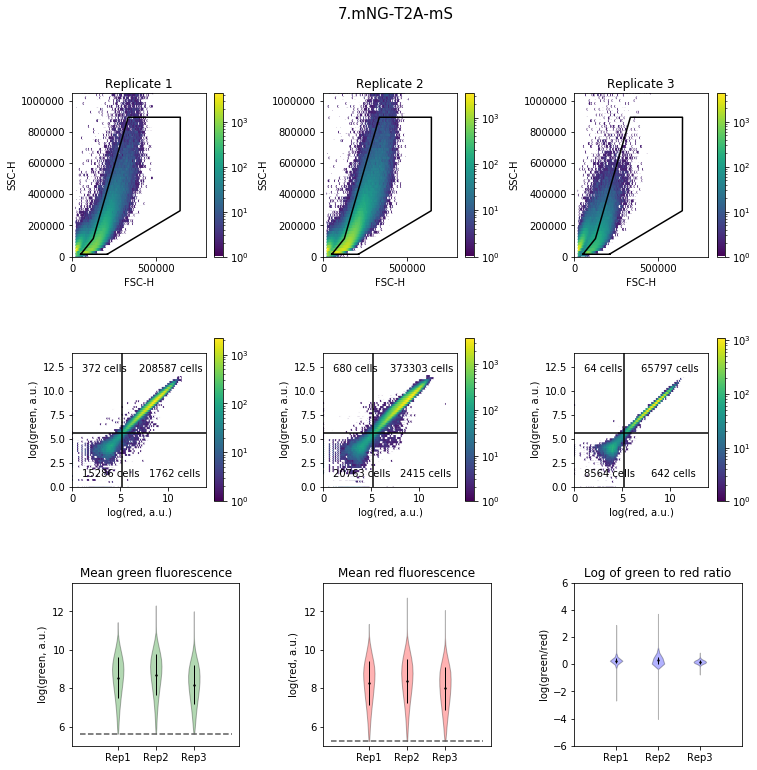

Supplement: S9 Fig — Top: Forward scatter and side scatter profiles of all three replicates. Density map color indicates event count. Events inside the polygon, defined as shown in S2 Fig, were considered live cells. Middle: Green and red fluorescence values for live cells for each replicate. Displayed numbers indicate total event count in a given quadrant. Cells in the upper right quadrant were retained for further analysis. Bottom: Mean fluorescence values and ratio of green to red fluorescence for each replicate. For each violin plot of single-cell values, the overlaid dot represents mean of the distribution and the error bars represent the standard deviation. Dashed lines represent fluorescence thresholds. (TIFF) [file pone.0281211.s012.tiff]

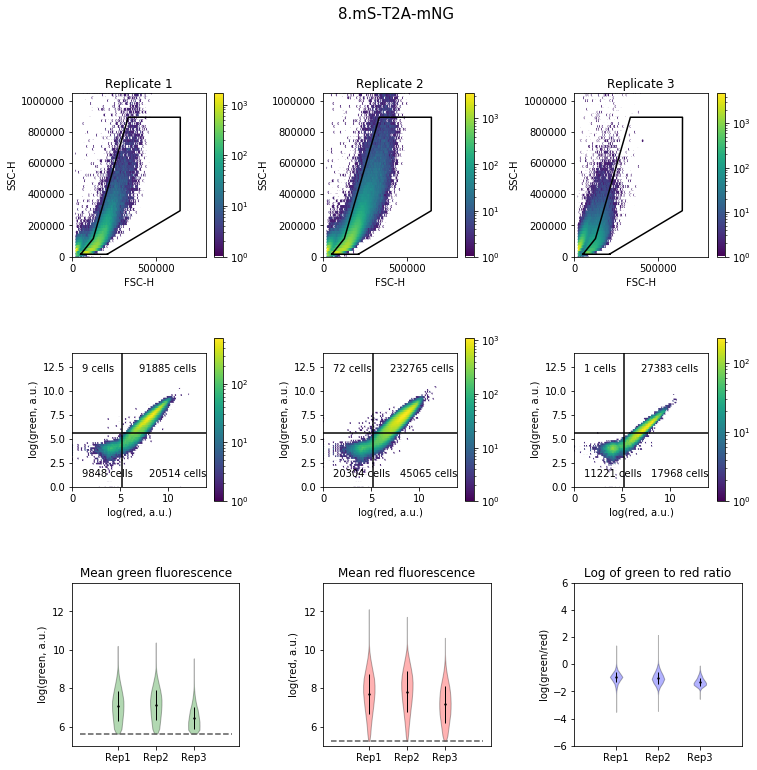

Supplement: S10 Fig — Top: Forward scatter and side scatter profiles of all three replicates. Density map color indicates event count. Events inside the polygon, defined as shown in S2 Fig, were considered live cells. Middle: Green and red fluorescence values for live cells for each replicate. Displayed numbers indicate total event count in a given quadrant. Cells in the upper right quadrant were retained for further analysis. Bottom: Mean fluorescence values and ratio of green to red fluorescence for each replicate. For each violin plot of single-cell values, the overlaid dot represents mean of the distribution and the error bars represent the standard deviation. Dashed lines represent fluorescence thresholds. (TIFF) [file pone.0281211.s013.tiff]

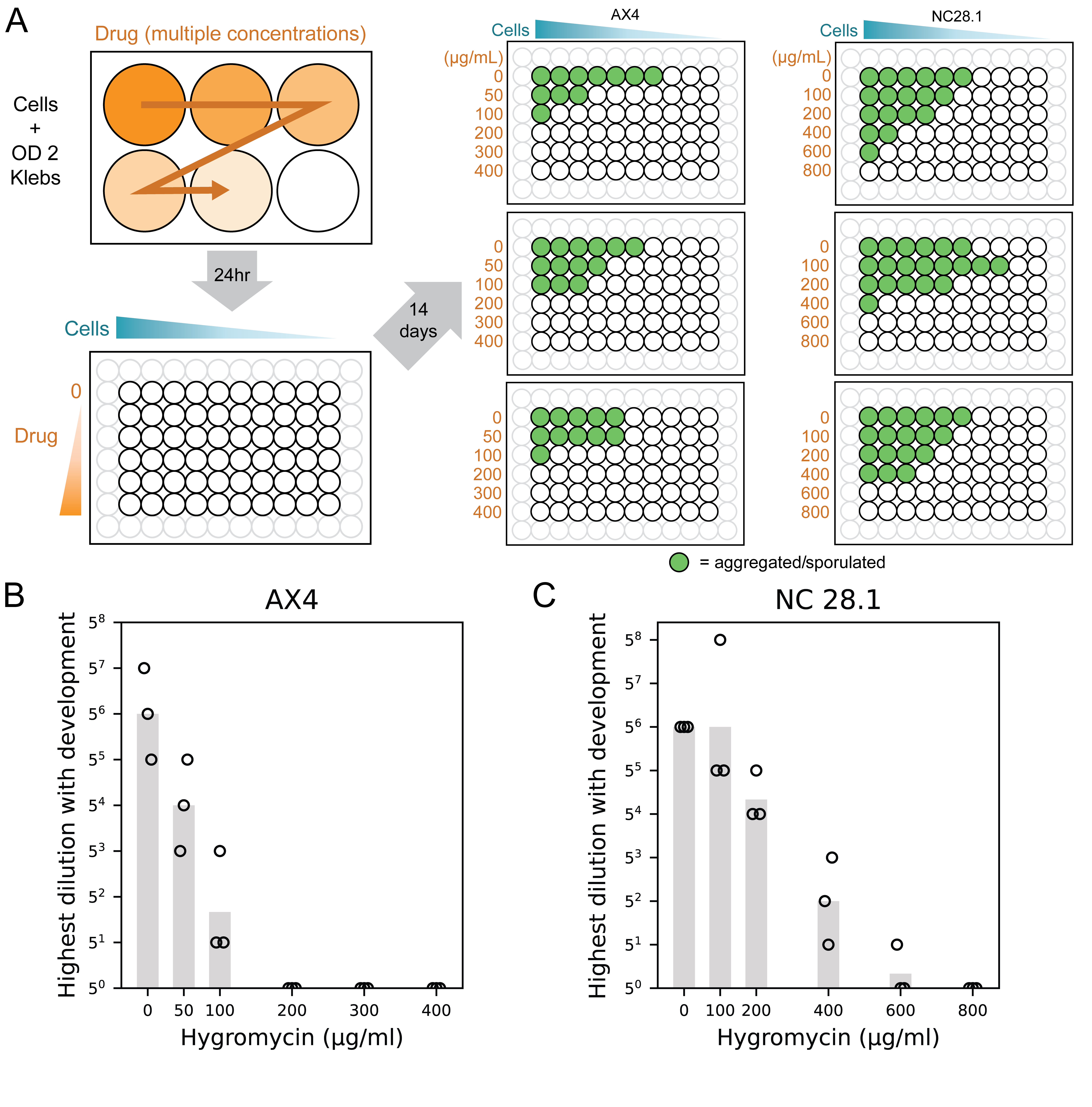

Supplement: S11 Fig — A: Diagram illustrating assay used to determine antibiotic susceptibility for different cell lines. Cells were grown with the indicated concentration of Hygromycin B Gold for 24 hours, then diluted and plated on SM agar. Wells were monitored for aggregation and development for two weeks after plating. At right, the final growth outcomes are shown for each replicate. B-C: Summarized assay results for AX4 (B) and NC28.1 (C). Each marker shows an independent experiment. (TIF) [file pone.0281211.s014.tif]

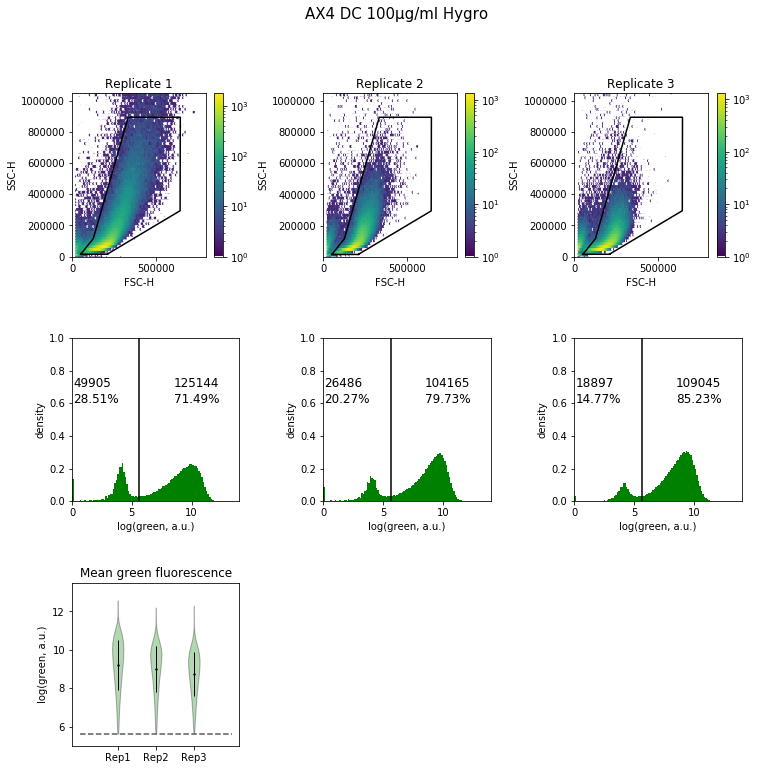

Supplement: S12 Fig — Top: Forward scatter and side scatter profiles of all three replicates. Events inside the polygon were considered live cells. Middle: Green fluorescence values for live cells for each replicate. Displayed numbers indicate the total event count and the percentage of events below or above the threshold. Cells above the fluorescence threshold were retained for further analysis. Bottom: Mean fluorescence values for each replicate. (TIFF) [file pone.0281211.s015.tiff]

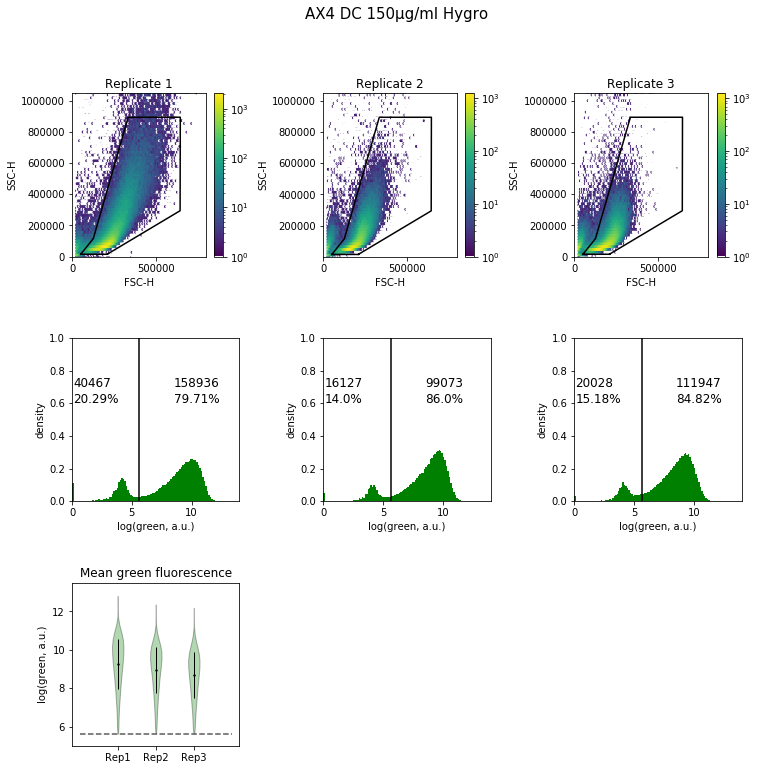

Supplement: S13 Fig — Top: Forward scatter and side scatter profiles of all three replicates. Events inside the polygon were considered live cells. Middle: Green fluorescence values for live cells for each replicate. Displayed numbers indicate the total event count and the percentage of events below or above the threshold. Cells above the fluorescence threshold were retained for further analysis. Bottom: Mean fluorescence values for each replicate. (TIFF) [file pone.0281211.s016.tiff]

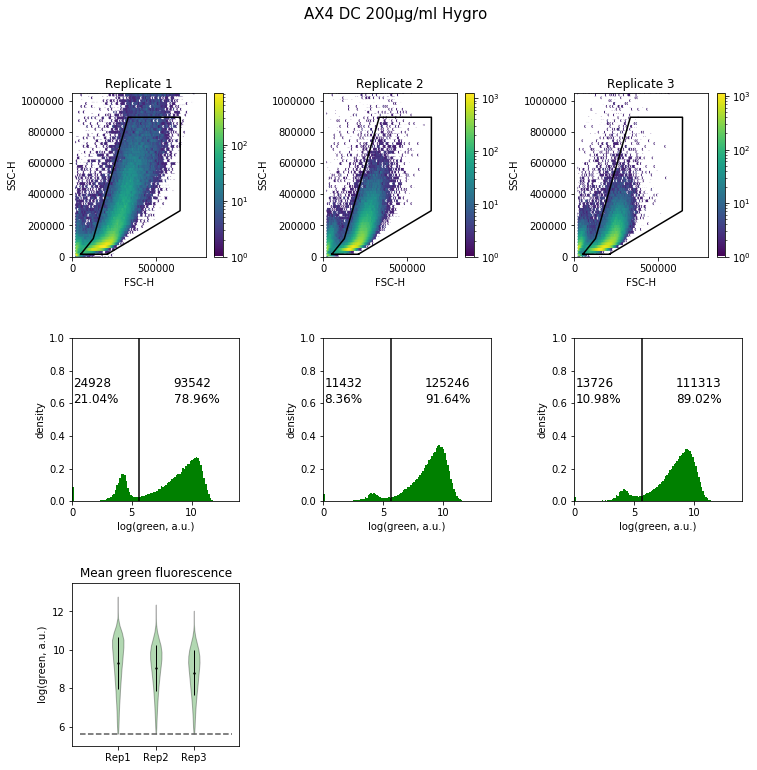

Supplement: S14 Fig — Top: Forward scatter and side scatter profiles of all three replicates. Events inside the polygon were considered live cells. Middle: Green fluorescence values for live cells for each replicate. Displayed numbers indicate the total event count and the percentage of events below or above the threshold. Cells above the fluorescence threshold were retained for further analysis. Bottom: Mean fluorescence values for each replicate. (TIFF) [file pone.0281211.s017.tiff]

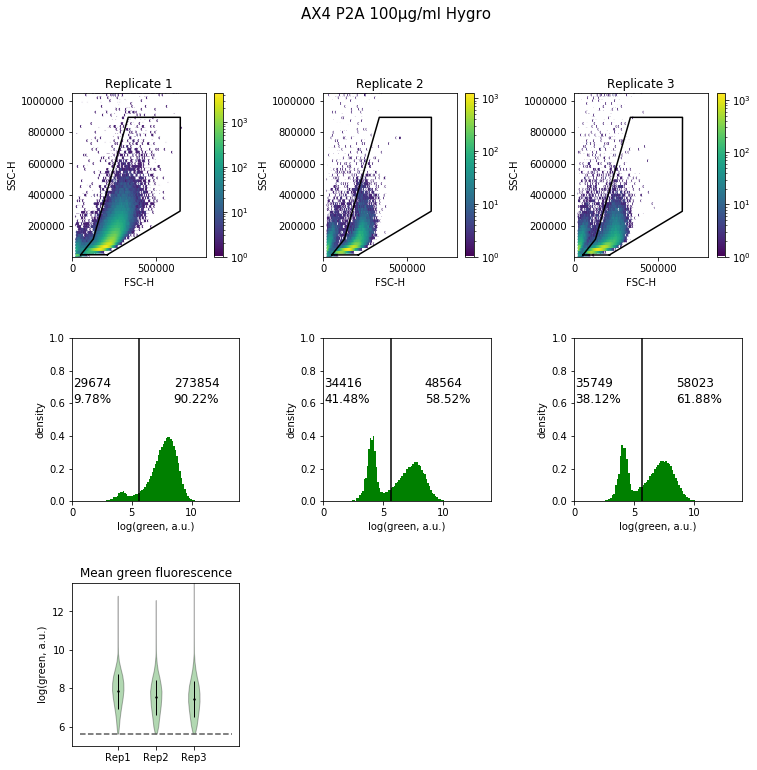

Supplement: S15 Fig — Top: Forward scatter and side scatter profiles of all three replicates. Events inside the polygon were considered live cells. Middle: Green fluorescence values for live cells for each replicate. Displayed numbers indicate the total event count and the percentage of events below or above the threshold. Cells above the fluorescence threshold were retained for further analysis. Bottom: Mean fluorescence values for each replicate. (TIFF) [file pone.0281211.s018.tiff]

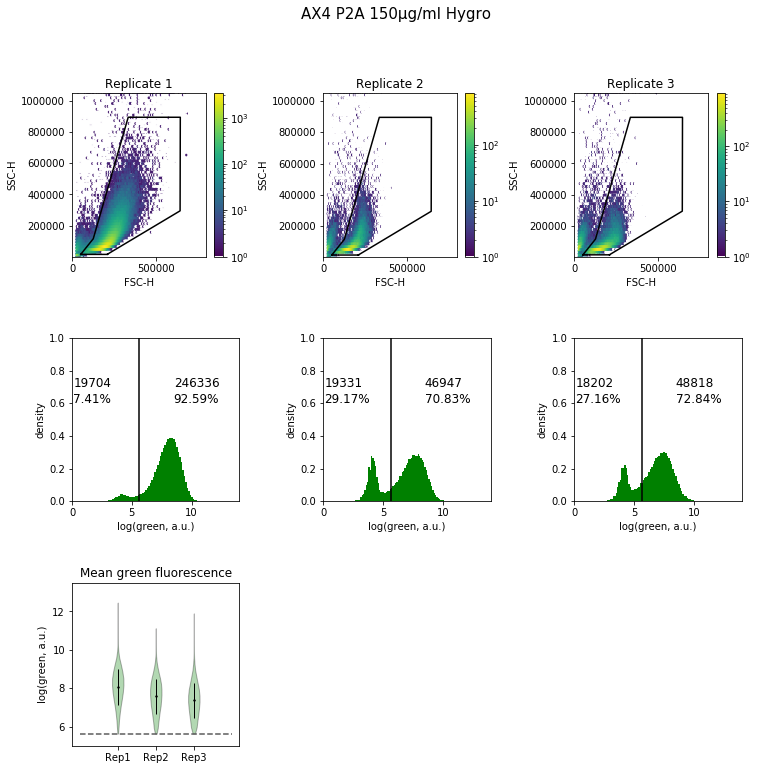

Supplement: S16 Fig — Top: Forward scatter and side scatter profiles of all three replicates. Events inside the polygon were considered live cells. Middle: Green fluorescence values for live cells for each replicate. Displayed numbers indicate the total event count and the percentage of events below or above the threshold. Cells above the fluorescence threshold were retained for further analysis. Bottom: Mean fluorescence values for each replicate. (TIFF) [file pone.0281211.s019.tiff]

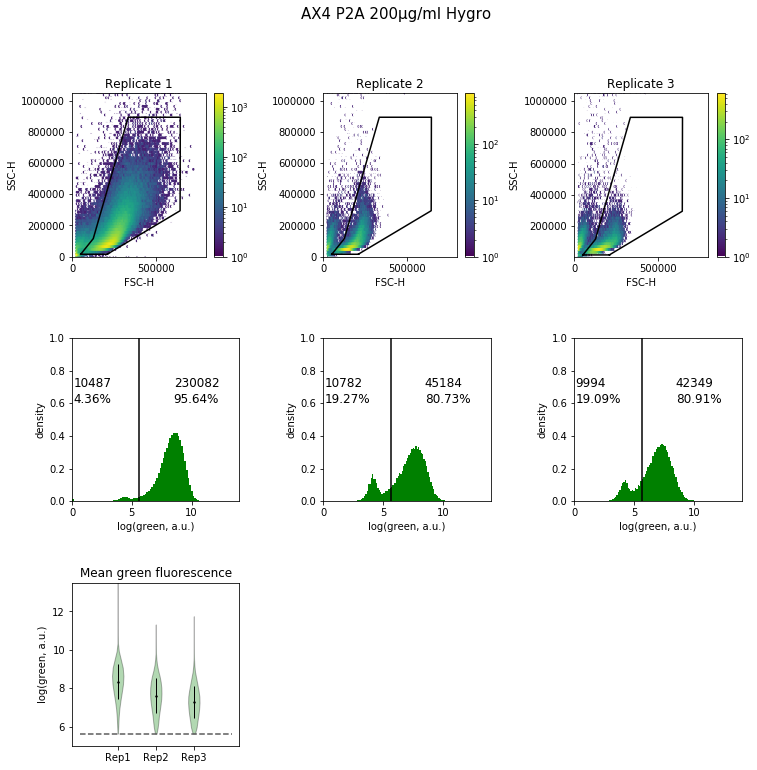

Supplement: S17 Fig — Top: Forward scatter and side scatter profiles of all three replicates. Events inside the polygon were considered live cells. Middle: Green fluorescence values for live cells for each replicate. Displayed numbers indicate the total event count and the percentage of events below or above the threshold. Cells above the fluorescence threshold were retained for further analysis. Bottom: Mean fluorescence values for each replicate. (TIFF) [file pone.0281211.s020.tiff]

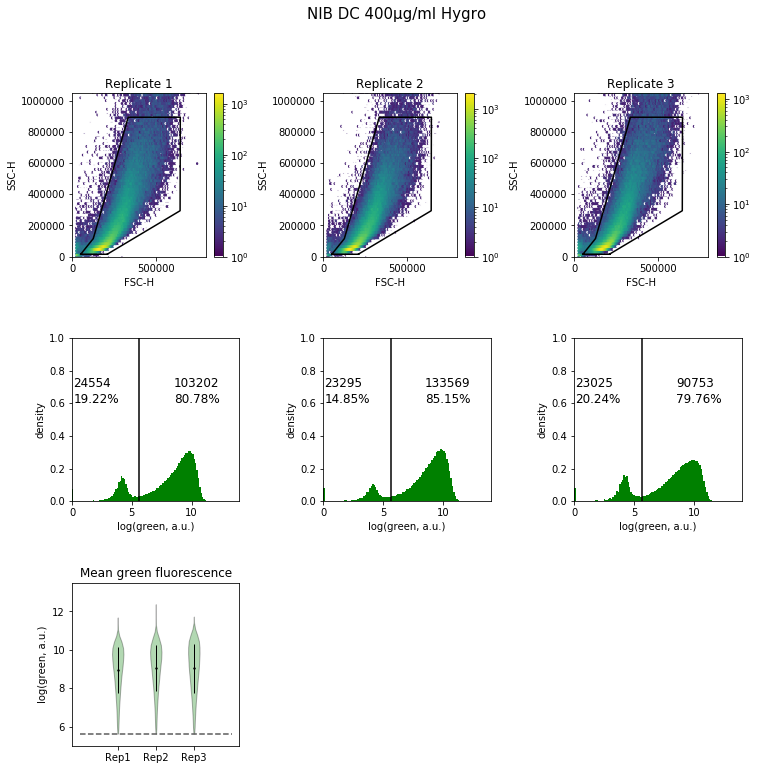

Supplement: S18 Fig — Top: Forward scatter and side scatter profiles of all three replicates. Events inside the polygon were considered live cells. Middle: Green fluorescence values for live cells for each replicate. Displayed numbers indicate the total event count and the percentage of events below or above the threshold. Cells above the fluorescence threshold were retained for further analysis. Bottom: Mean fluorescence values for each replicate. (TIFF) [file pone.0281211.s021.tiff]

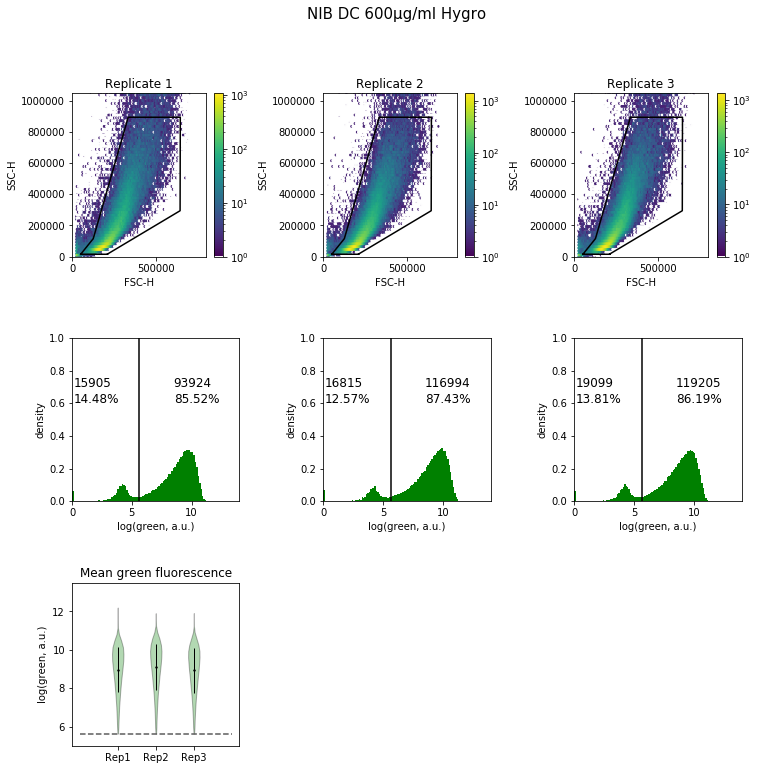

Supplement: S19 Fig — Top: Forward scatter and side scatter profiles of all three replicates. Events inside the polygon were considered live cells. Middle: Green fluorescence values for live cells for each replicate. Displayed numbers indicate the total event count and the percentage of events below or above the threshold. Cells above the fluorescence threshold were retained for further analysis. Bottom: Mean fluorescence values for each replicate. (TIFF) [file pone.0281211.s022.tiff]

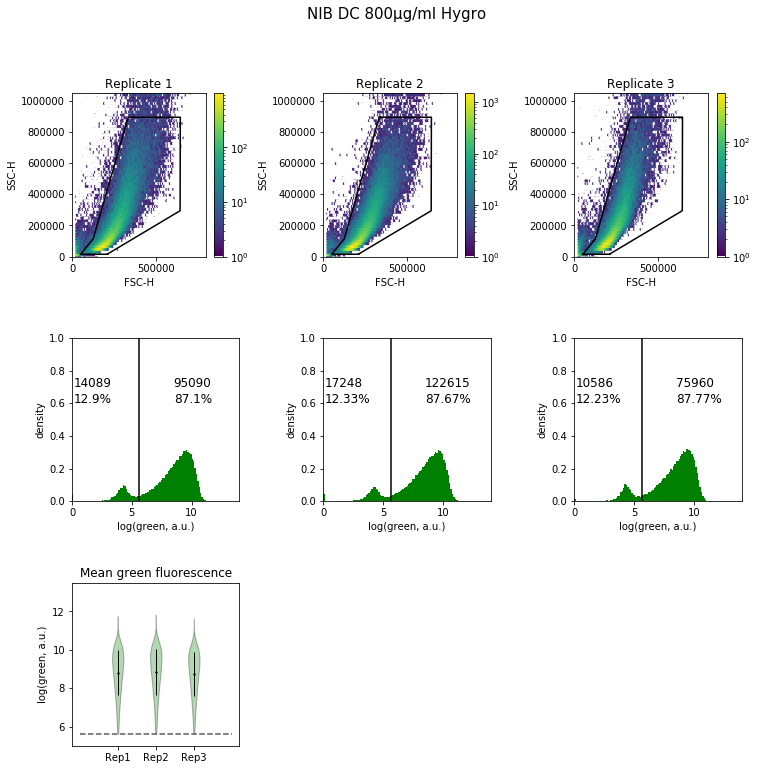

Supplement: S20 Fig — Top: Forward scatter and side scatter profiles of all three replicates. Events inside the polygon were considered live cells. Middle: Green fluorescence values for live cells for each replicate. Displayed numbers indicate the total event count and the percentage of events below or above the threshold. Cells above the fluorescence threshold were retained for further analysis. Bottom: Mean fluorescence values for each replicate. (TIFF) [file pone.0281211.s023.tiff]

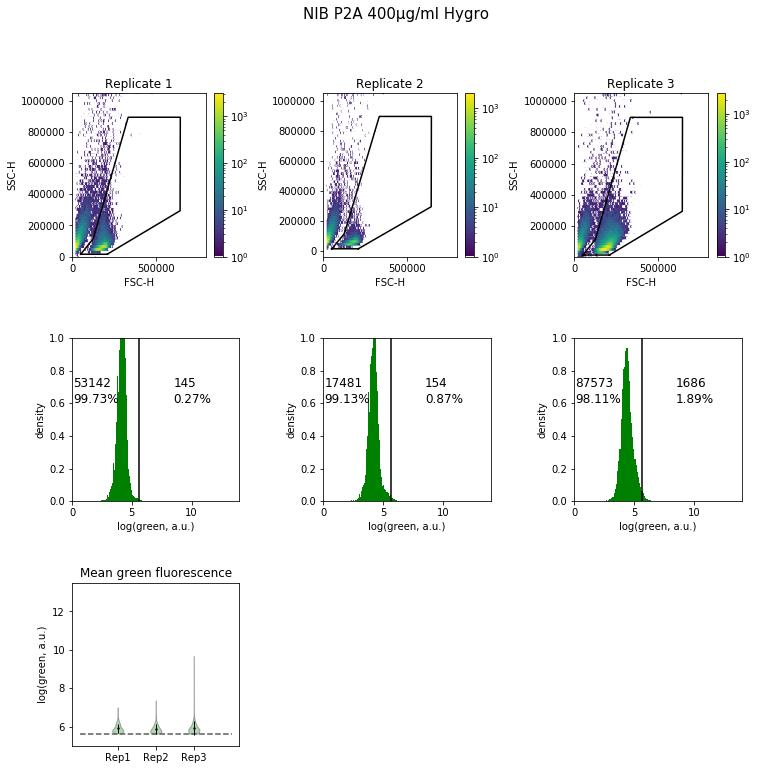

Supplement: S21 Fig — Top: Forward scatter and side scatter profiles of all three replicates. Events inside the polygon were considered live cells. Middle: Green fluorescence values for live cells for each replicate. Displayed numbers indicate the total event count and the percentage of events below or above the threshold. Cells above the fluorescence threshold were retained for further analysis. Bottom: Mean fluorescence values for each replicate. (TIFF) [file pone.0281211.s024.tiff]

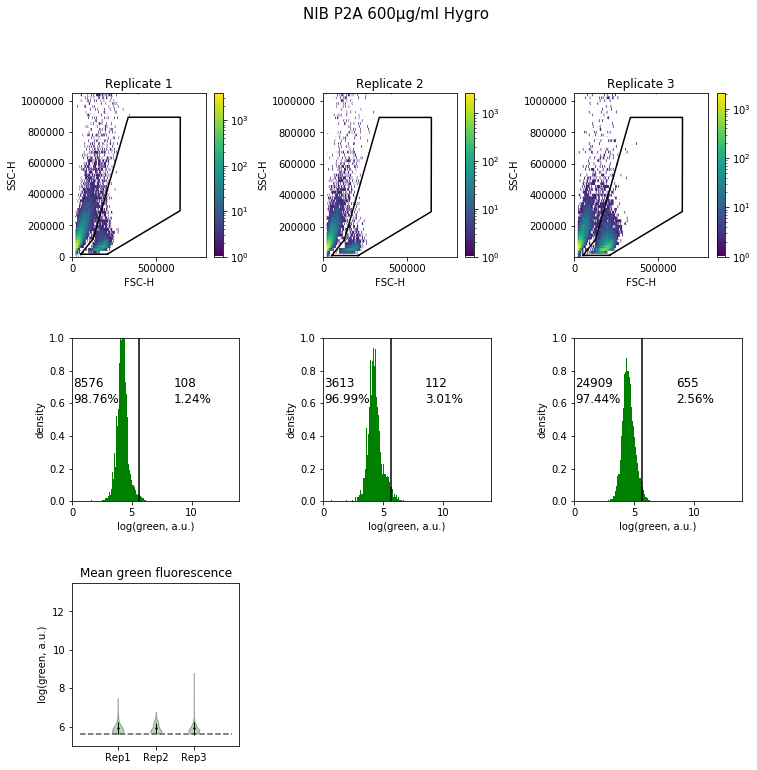

Supplement: S22 Fig — Top: Forward scatter and side scatter profiles of all three replicates. Events inside the polygon were considered live cells. Middle: Green fluorescence values for live cells for each replicate. Displayed numbers indicate the total event count and the percentage of events below or above the threshold. Cells above the fluorescence threshold were retained for further analysis. Bottom: Mean fluorescence values for each replicate. (TIFF) [file pone.0281211.s025.tiff]

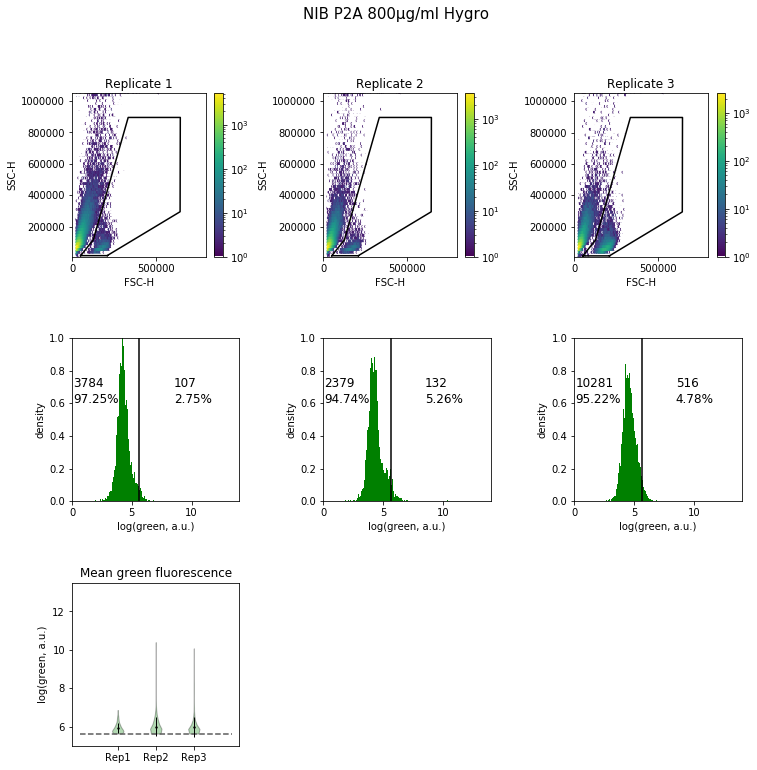

Supplement: S23 Fig — Top: Forward scatter and side scatter profiles of all three replicates. Events inside the polygon were considered live cells. Middle: Green fluorescence values for live cells for each replicate. Displayed numbers indicate the total event count and the percentage of events below or above the threshold. Cells above the fluorescence threshold were retained for further analysis. Bottom: Mean fluorescence values for each replicate. (TIFF) [file pone.0281211.s026.tiff]
