# Supplementary material for: Self-cleaving peptides for expression of multiple genes in Dictyostelium discoideum
Source: PLoS One. 2023 Mar 2;18(3):e0281211. doi: 10.1371/journal.pone.0281211 (PMC9980757; doi:10.1371/journal.pone.0281211)
Supplement: Attachment — Submitted filename: PLOS ONE Response to Reviewers.pdf [file pone.0281211.s027.pdf]

Below, reviewer comments are in *italics* and followed by our responses. In addition to the changes suggested by the reviewers, we have made small changes to the text and figures for clarity throughout the manuscript. Between the first submission and this revised submission, we have also added the following four references:

Ahem KG, Howard PK, Firtel RA. Identification of regions essential for extrachromosomal replication and maintenance of an endogenous plasmid in *Dictyostelium*. Nucleic Acids Research. 1988;16(14):6825–6837. doi:10.1093/nar/16.14.6825

Bloomfield G, Tanaka Y, Skelton J, Ivens A, Kay RR. Widespread duplications in the genomes of laboratory stocks of *Dictyostelium discoideum*. Genome Biology. 2008;9(4):R75. doi:10.1186/gb-2008-9-4-r75

Kuhn J, Lin Y, Devreotes PN. Using live-cell imaging and synthetic biology to probe directed migration in *Dictyostelium*. Frontiers in Cell and Developmental Biology. 2021;9:740205. doi:10.3389/fcell.2021.740205

Yurtsev E, Friedman J, Gore J. FlowCytometryTools: Version 0.4.5; 2015. Available from: https://zenodo.org/record/596118.

Response to Reviewer #1

The submitted work systematically establishes the usage of 2A peptides for the expression of dual reporters in Dictyostelium.

I deeply appreciate the effort the authors put into this manuscript to provide another useful tool for exploring different aspects of biology in Dictyostelium cells.
Well done on the statistics and flow cytometry data - that was a lot of work.

My comments and questions to the authors:

Figure 1:
Did you perform any protein level quantification before your loaded you gel to ensure equal amounts of protein was analyzed for all five constructs in your blot (BCA, Bradford or Precision Red - have not found it in the methods)? Or maybe you have a loading control like Tubulin? It would be a nice addition if you have the data already and just not shown in your manuscript since you do not use it for your quantification. It would further support your statement - at the moment it could also be that P2A looks better than T2A because you have loaded less sample.

We thank the reviewer for their helpful suggestion to further support our conclusions. We have repeated the western blot with a total protein loading control (No-Stain™ Protein Labeling Reagent, ThermoFisher Scientific), re-running all three original samples on the same gel to minimize any variability introduced between gels and blots and to highlight how reproducible the 2A cleavage rates are between independent transformations. In the revised experiment both P2A and T2A appear to have nearly complete cleavage, with mean cleavage rates of 99.6% (see Revised Figure 1B and C, New S1 Raw Images).

Figure 2/3:
How do your dual reporters work? Are they expressed from the same plasmid? Are both constructs (mCherry and mNeonGreen) using an act15 promoter and an act8 terminator?

It was not entirely clear to me from the text. Maybe you could explain this briefly in the main text or the figure legends?

To clarify these points, we have edited Figure 2 to clarify that all transcriptional units in this set of experiments use an actin15 promoter and an actin8 terminator. We have also edited the main text to include the sentences, “In all constructs, coding sequences for both fluorophores are present on the same plasmid. This single-plasmid approach ensures that in any given cell the two fluorophores are present with the same copy number. In our polycistronic constructs containing dual transcriptional units, both transcriptional units use the same promoter (actin15) and terminator (actin8) as the monocistronic constructs to minimize expression differences due to variable promoter strength.”

Do you think that maturation effects (10 min mNeon and 36 min mScarlet-I) could partially explain the better expression of constructs where mNeon is first? I always assumed that the oxidation step for the folding of fluorescent proteins makes the difference but you data might indicate that this is not the whole story.

We thank the reviewer for helping think through alternative mechanisms explaining our results. We agree that our data suggests multiple factors influence the observed brightness of both fluorophores in our expression constructs. However, we think that because both fluorophores are always brighter overall in constructs where mNeonGreen comes first it suggests we are looking at a difference in overall expression and not a difference in maturation behaviors.

Figure 4:
It might be a naive question, but I am not entirely sure after reading the text and figure legend: Is the construct driven by the act15 promoter or the CoA promoter? From our experience the CoA promoter is able to deliver enough protein to give cells G418 resistance under non-axenic conditions (We use it for knock outs and knock ins). We never texted act15 in this context, so I started wondering.

We thank the reviewer for pointing out this experimental detail we have now clarified in the text. In the experiments summarized in Figure 4, the mNeonGreen-P2A-HygroR construct is driven by an act15 promoter, while in the dual transcription unit plasmid mNeonGreen is driven by an act15 promoter and HygroR is driven by the coaA promoter. We have clarified the labels in Figure 4A and also included text in the associated legend to highlight the use of these different promoters. Note that as described in the Materials and Methods, the promoter and terminator sequences used in this set of experiments were derived from the pDM vectors described in Paschke, et al. and did not use the GoldenBraid system and its equivalent promoters and terminators.

We did conduct a preliminary trial where an mNeonGreen-P2A-NeoR construct was driven by a coaA promoter. We found that even with the coaA promoter we could not get sufficient expression of NeoR when it was downstream of P2A to have viable cells expressing the fluorescent protein upstream of the P2A sequence.

It is remarkable how resistant your NC4 isolate is towards hygromycin for DC conditions. Have you tested lower amounts of antibiotic for the screening when using the P2A construct to maybe obtain some clones?
Have you tested another non-axenic or axenic cell line? Or do you think the effect you see is unrelated to the ability to live in bacteria free liquid medium?

We agree that it is remarkable how poorly using a hygromycin resistance gene downstream of a P2A sequence works to drive expression of a fluorescent protein in the natural isolate NC28.1. We do see some dimly fluorescent cells at 400, 600, and 800 ug/ml of Hygromycin B Gold for all strains as shown in Supplementary Figures S21-23. At lower antibiotic concentrations we observed growth and proliferation of non-transfected cells as shown in Supplementary Figure S11, and while we tested an antibiotic concentration that allowed some growth and proliferation in NC28.1 (400 ug/ml) this did not lead to a notable increase in fluorescent cells (Supplementary Figure S21).

We did perform a pilot experiment with several additional natural isolate strains (NC34.1, NC105.1, NC85.2) at 800 ug/ml Hygromycin B Gold for all strains. We found that while all of them expressed mNeonGreen well in DC conditions, none of the natural isolate strains expressed the P2A construct as highly as AX4.

Three general question:

If I am correct you have always used extrachromosomal plasmids for your studies. Have you ever tried P2A or T2A in an integrating system like REMI or Act5? I was wondering if those strategies which depend on low copies of construct could also benefit from the 2A system or if the the decrease in expression you saw for the extrachromosomal plasmids would require further optimization for those (Please don't test it - I just ask out of curiosity).

We think this is a great idea and began cloning a P2A based construct into the act5 integration system, but we have yet to test it.

Did you ever tested more than 2 reporters at the same time? I am just wondering if ribosomal skipping is getting sequentially worse? (Please don't test this either)

We have not used more than 2 reporters, but a study conducted in mouse cells suggests that this is indeed the case and additional genes of interest separated by 2A sequences will have even lower expression (Liu, et al.).

Have you tested different promoters and terminators or did you directly decided on act15 and act8? I was just wondering about the effects of different transcription initiators and terminators (Curiosity again) in terms of expression drop when using 2A sequences.

All of the transcriptional units for fluorophores that we cloned used act15 and act8. We did use the native coaA promotoer and mhcA terminator from the pDM vectors described in Paschke, et al. to drive the hygromycin resistance gene in our dual-transcription unit construct study shown in Figure 4, and we have updated Figure 4 to clarify which constructs used which promoter and terminator. We have not yet explored alternative initiators and terminators for fluorescent proteins.

Good luck for the final submission!

Response to Reviewer 2

The present manuscript by Sgro and colleagues is a technical report of the establishment and quantification of polycistronic expression for the model organism Dictyostelium. They demonstrate that 2A viral peptides are capable of mediating effective cleavage between first and second protein and that gene order effects are milder for P2A. This is an interesting study

that sheds further light into the genetic toolkit of Dictyostelium. These data are generally of high quality, and the manuscript is within the remit of PLoS One. However, there are several concerns with the interpretation of the data.

Major comments:
1- The authors show convincing evidence that 2A peptides are capable of producing unlinked protein products. However, even with P2A, which demonstrated the highest 99.6% of cleavage efficiency, mNG fluorescence is enriched at centre of the cell enough to identify nuclear shape. Therefore, it seems that mNG data do not fully support the conclusion. Especially, it is possible that the present cleavage efficiencies do not reflect the dynamic behaviour of the protein in the cells. The recent manuscript presented by Hillmann and colleagues showed viral 2A sequences function in the expression of multiple enzymes in Dictyostelium, thus precise quantification of the efficiency is an important piece in this study.

2- Related to the above point, it would be good to quantify the nuclear to cytoplasmic ration of mNG using the imaging data. What about the impact of cleavage efficiency calculated by the different approaches? The authors should also show whether mNG without H2B distributes uniformly throughout the cell. Might be enough to be shown in the Supplementals.

We thank the reviewer for their careful consideration of our data and suggestion of additional controls to strengthen our conclusions. Both of these major concerns are dependent on how mNeonGreen is distributed in the cell when expressed alone. We agree that there is visible nuclear enrichment of mNeonGreen even when used with the high cleavage efficiency 2A sequences P2A and T2A in Figure 1A. To explore the source of this nuclear enrichment, we conducted a new set of control experiments where we expressed mNeonGreen alone without any type of nuclear localization or escape sequence as this mirrors how it is constructed in our 2A and dual reporter constructs. In these controls, we observe a slight enrichment of green fluorescence in the center of the cell (likely the nucleus). These data are shown in the new Supplemental Figure S1. These data support the idea that the spatial distribution of mNeonGreen observed in Figure 1A is not inconsistent with the high rate of cleavage observed in the western blot shown in Figure 1B and quantified in Figure 1C.

3- It should be useful to integrate an extra figure just giving a brief overview of the created plasmids. The community should further validate the newly created resources rather than only the lab of the authors, so it would be helpful if you could briefly explain how to replace the fluorescent proteins with other target genes of interest. How would that be if there are three or four target genes? How would you ligate them together? Since the new genetic tools are a major part of the paper, it would further improve the manuscript.

We agree with the reviewer that reproducibility and sharing of new resources are important. To minimize variability by creating a new plasmid or expression approach for this paper, we used plasmid constructs and cloning systems previously developed by other labs. For the studies in Figures 1-3, we used the GoldenBraid system described in Kundert, et al., and for Figure 4 we used the pDM vectors described in Paschke, et al.. To enable further validation and widespread use of our 2A sequences and fluorophores by the community, GoldenBraid plasmids we created for the 2A sequences and the codon-optimized fluorescent proteins in this study are available from the Dicty Stock Center.

Minor comments:
1- Line 15, Maybe the authors should explain that knock-in is an established technique to express two components at a similar level. Two identical genomic regions of chromosome 2

duplication in AX4 can be used to achieve almost the same level of expression. Highly efficient knock-in methods have been reported for Dictyostelium.

We have included the following text: “In the standard axenic lab strains, known genomic duplications allow for the knock-in of different genes at near-identical genomic loci to achieve similar levels of expression (Bloomfield, et al.).”

2- The authors state that combining the coding sequences of two proteins into a single transcript leads to notable decreases in expression levels with strain-dependent manner. To avoid confusion, it would be important to clarify if this phenomenon is common to various strains of Dictyostelium or just the specific difference between AX4 and NC28.1. These strains show originally many differences, including axenic or non-axenic strains, growth rates, drug sensitivity and mating types.

We thank the reviewer for suggesting this modification to clarify our findings. As described in our response to Reviewer 1, we did perform pilot experiments on several non-axenic strains, but only explored the difference between AX4 and NC28.1 in a comprehensive manner. We have clarified this in the text by replacing “strain-dependent” with specific mention of the two tested strains.

3- The authors create an interesting genetic toolkit, while its specific application is still ambiguous. Concerted expression of CRY2/CIBN is an interesting application, however quantitative control of protein levels would not be simple by P2A system. A slightly more extensive discussion of the applications specific to the system would be helpful.

We appreciate this suggestion to improve our manuscript. We have edited both the introduction and the conclusion to better integrate applications where closely linked expression levels would be helpful.

4- Line 154, 90C --> 90°C

5- Line 160, 4C --> 4°C

6- Fig.3 legend, *: p¡0.05 --> *: p>0.05

We thank the reviewer for pointing out these formatting errors. They have been corrected.
